# The frequency of influenza and bacterial coinfection: a systematic review and meta‐analysis

**DOI:** 10.1111/irv.12398

**Published:** 2016-06-24

**Authors:** Eili Y. Klein, Bradley Monteforte, Alisha Gupta, Wendi Jiang, Larissa May, Yu‐Hsiang Hsieh, Andrea Dugas

**Affiliations:** ^1^ Department of Emergency MedicineJohns Hopkins UniversityBaltimoreMDUSA; ^2^ Center for Disease Dynamics, Economics & PolicyWashingtonDCUSA; ^3^ Eastern Virginia Medical SchoolNorfolkVAUSA; ^4^ Allegheny General HospitalPittsburghPAUSA; ^5^ Department of Emergency MedicineThe George Washington UniversityWashingtonDCUSA

**Keywords:** antibiotic resistance, bacterial coinfection, influenza, meta‐analysis, MRSA, *Streptococcus Pneumoniae*

## Abstract

**Aim:**

Coinfecting bacterial pathogens are a major cause of morbidity and mortality in influenza. However, there remains a paucity of literature on the magnitude of coinfection in influenza patients.

**Method:**

A systematic search of MeSH, Cochrane Library, Web of Science, SCOPUS, EMBASE, and PubMed was performed. Studies of humans in which all individuals had laboratory confirmed influenza, and all individuals were tested for an array of common bacterial species, met inclusion criteria.

**Results:**

Twenty‐seven studies including 3215 participants met all inclusion criteria. Common etiologies were defined from a subset of eight articles. There was high heterogeneity in the results (*I* ^*2*^ = 95%), with reported coinfection rates ranging from 2% to 65%. Although only a subset of papers were responsible for observed heterogeneity, subanalyses and meta‐regression analysis found no study characteristic that was significantly associated with coinfection. The most common coinfecting species were *Streptococcus pneumoniae* and *Staphylococcus aureus*, which accounted for 35% (95% CI, 14%–56%) and 28% (95% CI, 16%–40%) of infections, respectively; a wide range of other pathogens caused the remaining infections. An assessment of bias suggested that lack of small‐study publications may have biased the results.

**Conclusions:**

The frequency of coinfection in the published studies included in this review suggests that although providers should consider possible bacterial coinfection in all patients hospitalized with influenza, they should not assume all patients are coinfected and be sure to properly treat underlying viral processes. Further, high heterogeneity suggests additional large‐scale studies are needed to better understand the etiology of influenza bacterial coinfection.

## What this adds to existing literature

Clinical treatment of influenza presents difficulties because of significant uncertainty regarding the probability of bacterial coinfection. Despite this uncertainty, the frequency of overall coinfection in influenza patients is still poorly characterized. This meta‐analysis increases understanding of the likelihood that patients hospitalized with influenza also have a bacterial coinfection; however, the variability in results suggests that physicians should ensure that appropriate cultures are taken to minimize the overuse of antibiotics.

## Introduction

Influenza causes widespread annual epidemics infecting up to 20% of the population and resulting in significant morbidity and mortality.^1^ Coinfecting bacterial pathogens are a major cause of that morbidity and mortality and are associated with both pandemic and seasonal influenza virus illness.^2^ Lung tissue samples from the 1918 influenza pandemic suggest that the majority of the estimated 20–60 million deaths were from bacterial infections rather than from direct effects of the virus.^3^ In seasonal epidemics, influenza bacterial coinfection is associated with increases in hospital admissions,^4^, ^5^ more severe symptoms,^6^ and increases in mortality.^7^ Viral damage to the epithelial lining of the respiratory tract is believed to facilitate establishment of bacterial infections.^8^, ^9^, ^10^ However, other factors, such as changes in airway function, up‐regulation and exposure of receptors, dampening of the immune response, or enhancement of inflammation may also play a role.^11^

Clinically, it can be difficult to identify influenza patients experiencing bacterial coinfections, given the substantial symptom overlap of influenza and bacterial infections. Identification of coinfected patients and coinfecting pathogen enables clinicians to initiate appropriate antibiotic therapy and improve patient outcomes.^12^ While prior studies have examined the frequency of *select* bacterial species in influenza cases,^13^, ^14^ particularly the presence of methicillin‐resistant *S. aureus* (MRSA),^15^, ^16^, ^17^, ^18^ the frequency of overall coinfection in influenza patients is still poorly characterized. We undertook a systematic review to determine the frequency of bacterial coinfections in patients with laboratory confirmed influenza and to identify the most common coinfecting bacterial species.

## Methods

We conducted a systematic review, which is reported in accordance with PRISMA guidelines,^19^ to determine the frequency of bacterial coinfection among individuals with laboratory confirmed influenza. Inclusion was restricted to studies of humans in which all individuals had laboratory confirmed influenza, and all individuals were tested for an array of common bacterial species. Studies reanalyzing prior published data were excluded. There were no limitations based on participant age or the location of participant recruitment (i.e., community, outpatient, hospital). Coinfection was assumed to be any acute bacterial infection identified in respiratory secretions, sputum, or sterile site (e.g., bacteremia). We restricted results to publications in English published after January 1982. To avoid analyses of historic samples, particularly related to the influenza pandemic of 1968, studies using data collected prior to 1972 were excluded. Case reports, defined as studies with a sample size of fewer than 10 individuals, were excluded, but no other limitations based on study design were imposed.

### Literature search

We performed a systematic search of MeSH, Cochrane Library, Web of Science, SCOPUS, EMBASE, and PubMed for publications in August 2014. The search terms included influenza, bacterial infection, bacterial coinfection, bacterial pathogens, bacteremia, bacterial–viral infection, coinfection, secondary infection, mixed infection, concomitant infection, H1N1, swine influenza, bird flu, gripe, pandemic influenza, seasonal influenza, influenza virus A H1N1, and avian influenza. The complete search strategy, which was completed in consultation with a research librarian, is detailed in Table S1.

### Selection of studies

Two authors (BM, AG) independently screened the title and abstract of all the search‐returned publications to determine whether they met study criteria. The full text of all studies meeting the criteria and those for which a conclusion could not be made were reviewed independently by the two authors. Disagreements were resolved through consultation with a third party.

### Data extraction

A structured data extraction form was used to collect data elements of each study into a Microsoft Excel worksheet. Two authors (BM, AG) extracted study data from all included publications independently, and results were then compared. Differences were resolved by consensus. Information extracted included: study design (i.e., prospective, retrospective), location of the study, study size, year of enrollment, study enrollment setting (i.e., intensive care unit [ICU], hospital, or emergency department [ED]), influenza strain (A, A pH1N1, B, all), participant age, bacterial collection method (sputum, blood, bronchial alveolar lavage [BAL]), method of bacterial detection (stain, culture, polymerase chain reaction [PCR], antibody), bacterial species evaluated, and bacterial species identified. In cases where only a percentage or subject number was published, its counterpart was calculated for analysis in the current review. Sources of data were carefully reviewed by BM, AG, EK, and studies reporting already included data were excluded (the study with the earlier publication date was considered the primary study, and all others excluded).

### Assessment of bias

The potential bias of each study was assessed using the Quality Assessment Tool for Quantitative Studies developed by the National Collaborating Centre for Methods and Tools.^20^ This tool was selected for its comprehensive ability to assess the methodological quality of non‐randomized studies and has shown good reliability and validity.^21^, ^22^ A 3‐point scale was used for the following criteria: selection bias, study design, confounders, blinding, data collection methods, and study withdrawals. A global rating of “strong” was awarded for 4 “strong” ratings and no “weak” ratings, “moderate” for less than four “strong” and one “weak,” and “weak” for two or more “weak” ratings. Each study was independently evaluated by two authors, and discrepancies regarding bias assessment were resolved by consensus. Funnel plots and calculation of Egger's test of asymmetry were also used to assess biases such as publication and small‐study effects.^23^

### Data analysis

The primary outcome was the proportion of bacterial coinfection. Coinfection was defined as the number of cases with a confirmed bacterial coinfection in all tested cases of patients with laboratory confirmed influenza. Because of differences between studies, we analyzed combined data on coinfection frequencies using the DerSimonian‐Laird method in the metaphor package,^24^ a meta‐analysis package for R.^25^ Heterogeneity was quantified using the *I* ^*2*^ statistic.^26^ Least‐squares meta‐regressions were performed to investigate the effect of differences in *a priori* defined trial‐level characteristics on the frequency of coinfection.^27^ These included: (i) age of the participants; (ii) study enrollment setting; (iii) year of enrollment; (iv) retrospective or prospective study design; (v) study size; (vi) bacterial collection method (BAL versus other); and (vii) method of bacterial detection. For bacterial detection, we examined the types of tests used to detect bacteria individually as well as the total number of tests used. To investigate the heterogeneity between studies and the influence of studies on the results, we performed a leave‐one‐out analysis as well as used Cook's distances to group the most heterogeneous studies. For species‐level analysis, only studies providing the numbers or percentages of each bacterial coinfecting pathogen were included. We included all pathogens in cases where more than one bacterial pathogen was found.

## Results

### Study screening and selection

**Figure 1:**
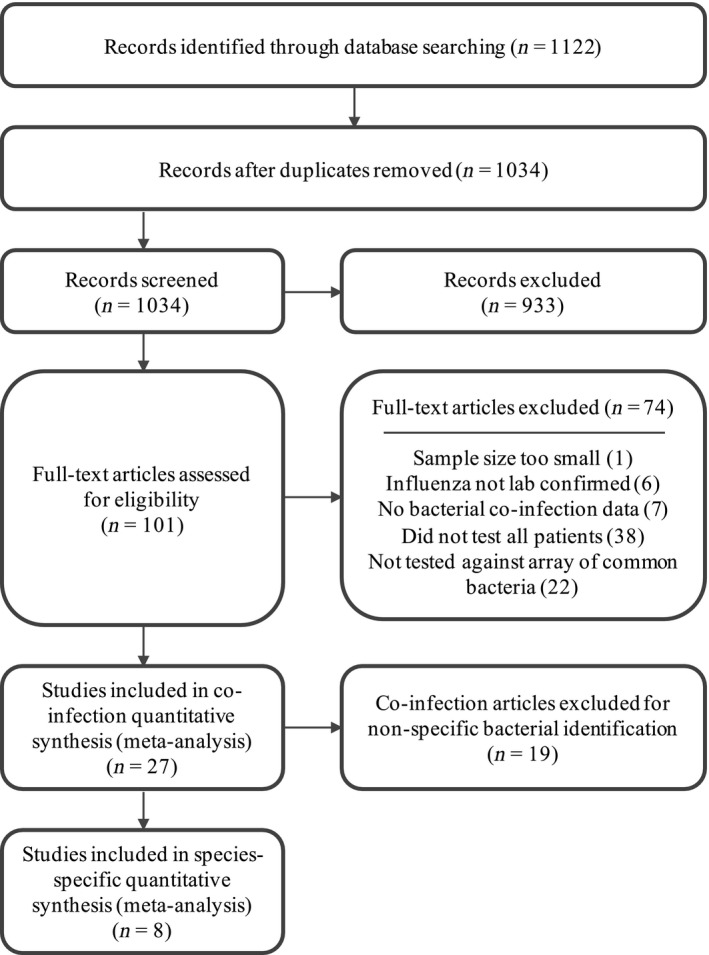
Flow diagram of study selection.

The initial literature search yielded 1122 references, which was reduced to 1034 after removing duplicates. Following initial abstract review, 101 articles remained. The full‐text review resulted in the exclusion of an additional 74 articles. A total of 27 articles encompassing 3215 patients met all the inclusion criteria and were included in the final analysis.^28^, ^29^, ^30^, ^31^, ^32^, ^33^, ^34^, ^35^, ^36^, ^37^, ^38^, ^39^, ^40^, ^41^, ^42^, ^43^, ^44^, ^45^, ^46^, ^47^, ^48^, ^49^, ^50^, ^51^, ^52^, ^53^, ^54^ Of those 27, only eight studies, with 334 patients, provided the numbers or percentages of each bacterial coinfecting pathogen.^28^, ^32^, ^33^, ^34^, ^38^, ^43^, ^46^, ^47^ Thus, these eight studies formed the basis for identifying the most common coinfecting bacterial pathogens (Figure 1).

### Study characteristics

All 27 included studies were observational, of which 13 (1218 patients) were prospective studies,^30^, ^32^, ^33^, ^36^, ^40^, ^41^, ^43^, ^44^, ^48^, ^49^, ^50^, ^51^, ^52^ and 14 (1997 patients) were retrospective analyses (Table 1). The majority (21) of studies were cohort studies, and the rest were case–control studies. Fifteen of the studies (1885 patients) began enrollment in 2009 or later,^28^, ^32^, ^33^, ^34^, ^35^, ^37^, ^39^, ^41^, ^42^, ^44^, ^46^, ^48^, ^49^, ^53^, ^54^ and of these, all but one was specifically focused on the 2009 H1N1 pandemic strain. Most studies enrolled only adults, although the lower age cutoffs varied (e.g., some studies considered adults older than 14 while others used 21). Only seven studies (1214 patients) focused on young children or newborns,^29^, ^31^, ^38^, ^46^, ^49^, ^51^, ^54^ and two studies (504 patients) included both children and adults.^42^, ^53^ Severity of illness varied among studies, but all were focused on hospitalized patients, suggesting a greater than average severity. Eleven of the studies (1007 patients) focused exclusively on patients initially enrolled in an intensive care unit (ICU),^32^, ^34^, ^35^, ^38^, ^39^, ^41^, ^42^, ^44^, ^46^, ^47^, ^51^ 10 enrolled non‐ICU hospitalized patients (973 patients),^30^, ^33^, ^36^, ^37^, ^40^, ^43^, ^45^, ^49^, ^53^, ^54^ 3 enrolled patients in the emergency department (ED; 135 patients),^28^, ^48^, ^50^ and 2 enrolled patients in a mix of inpatient and outpatient settings (1100 patients).^29^, ^52^ The median sample size was 51 (IQR, 18·5–101), and the mean was 119 (SD: 203), which was driven by three large studies^29^, ^44^, ^53^ that contributed 1958 (61%) of the total number of patients included in this analysis.

**Table 1:** Study characteristics

Study	Country (study)	Enrollment Year	Study Design	Setting	Participant Age Class	Sample Size	Bacterial CoInfection (%)
Ahn S, *et al*., 2011^28^	South Korea	2009	Retrospective (cohort)	ED	Adult	60	26·7
Bender JM, *et al*., 2010^29^	USA	2004	Retrospective (cohort)	Mixed	Pediatric	833	1·9
Bjarnason A, *et al*., 2012^30^	Iceland	2008	Prospective (cohort)	Hospital	Adult	22	13·6
Carr SB, *et al*., 2012^31^	USA	2002	Retrospective (cohort)	Mixed	Pediatric	107	2·8
Choi S‐H, *et al*., 2012^32^	South Korea	2010	Prospective (cohort)	ICU	Adult	12	33·3
Cordero E, *et al*., 2011^33^	Spain	2009	Prospective (cohort)	Hospital	Adult	51	11·8
Cuquemelle E, *et al*., 2011^34^	France	2009	Retrospective (case–control)	ICU	Adult	103	46·6
Dave BM, 2014^35^	India	2012	Retrospective (cohort)	ICU	Adult	34	35·3
Falsey AR, *et al*., 2012^36^	USA	2008	Prospective (cohort)	Hospital	Adult	90	12·2
Guervilly C, *et al*., 2010^37^	France	2009	Retrospective (cohort)	Hospital	Adult	99	11·1
Hon KL, *et al*., 2008^38^	China	2003	Retrospective (cohort)	ICU	Pediatric	13	15·4
Ingram PR, *et al*., 2010^39^	Australia	2009	Retrospective (case–control)	ICU	Adult	17	5·9
Johansson N, *et al*., 2010^40^	Sweden	2004	Prospective (cohort)	Hospital	Adult	14	50·0
Lopez‐Delgado J, *et al*., 2013^41^	Spain	2009	Prospective (cohort)	ICU	Adult	60	25·0
Malato L, *et al*., 2011^42^	France	2009	Retrospective (cohort)	ICU	Both	24	25·0
Marcos MA, *et al*., 2006^43^	Spain	2003	Prospective (cohort)	Hospital	Adult	16	25·0
Martin‐Loeches I, *et al*., 2011^44^	Spain	2009	Prospective (cohort)	ICU	Adult	645	17·5
Mermond S, *et al*., 2010^45^	France	2006	Prospective (cohort)	Hospital	Adult	26	65·4
Nguyen T, *et al*., 2012^46^	USA	2009	Retrospective (cohort)	ICU	Pediatric	66	51·5
Schnell D, *et al*., 2013^47^	France	2007	Retrospective (cohort)	ICU	Adult	13	15·4
Sohn CH, *et al*., 2013^48^	South Korea	2009	Prospective (cohort)	ED	Adult	59	25·4
Torres JP, *et al*., 2012^49^	Chile	2009	Prospective (cohort)	Hospital	Pediatric	27	25·9
Van Gageldonk‐Lafeber AB, *et al*., 2013^50^	Netherlands	2007	Prospective (cohort)	ED	Adult	16	18·8
Vieira RA, *et al*., 2003^51^	Brazil	1999	Prospective (case–control)	ICU	Pediatric	20	30·0
von Baum H, *et al*., 2011^52^	Germany	2002	Prospective (case–control)	Mixed	Adult	160	21·3
Yan XX, *et al*., 2011^53^	China	2009	Retrospective (case–control)	Hospital	Both	480	19·0
Zhang Q, *et al*., 2012^54^	China	2009	Retrospective (case–control)	Hospital	Pediatric	148	25·7

ED, Emergency Department; ICU, Intensive Care Unit.

### Assessment of bias

**Figure 2:**
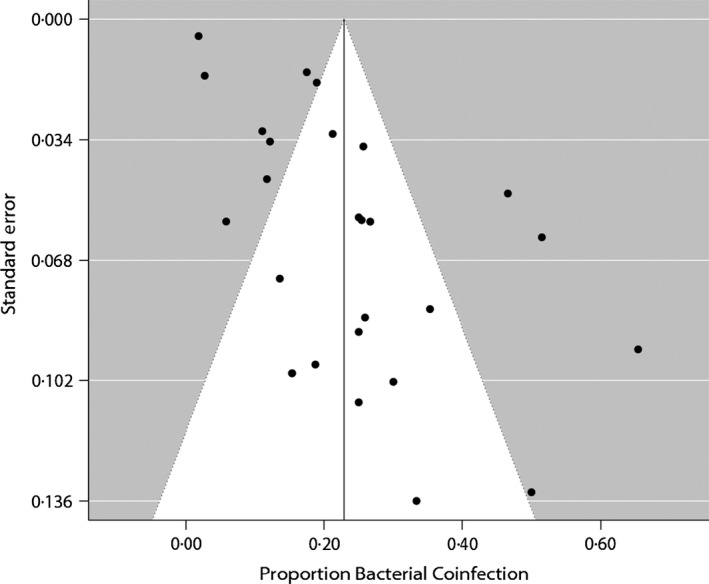
Funnel plot of each study's standard error (*y*‐axis) against each study's frequency of bacterial coinfection in laboratory confirmed hospitalized patients. Because small studies have less precision and large studies have more, scatter should form an inverted funnel when there are no systematic missing studies. The line indicates the overall mean frequency of coinfection (23%). The funnel plot appears asymmetric. Egger's test of asymmetry was significant for bias (*P *= 0·0004).

The average quality of the studies was moderate, with the most common quality issues being related to study design, selection biases, and data collection (Table S2). While studies were generally representative of the targeted population, most studies did not report the percentage of patients that agreed to participate. This lack of detail did not allow us to eliminate the possibility of selection bias in the study cohorts. Studies were also not fully clear on the reliability of the tools used for data collection, which made it difficult to eliminate this as a potential source of bias in the strains detected. However, results from the bias assessment suggest that the largest potential source of bias was the fact that all the studies were observational studies, and most were small cohort studies. A funnel plot appeared asymmetrical (Figure 2), suggesting statistical heterogeneity; in particular, there seems to have been a lack of smaller studies with lower rates of bacterial coinfection. Egger's test of asymmetry was also significant for bias (*P *= 0·0004).

### Bacterial coinfection

**Figure 3:**
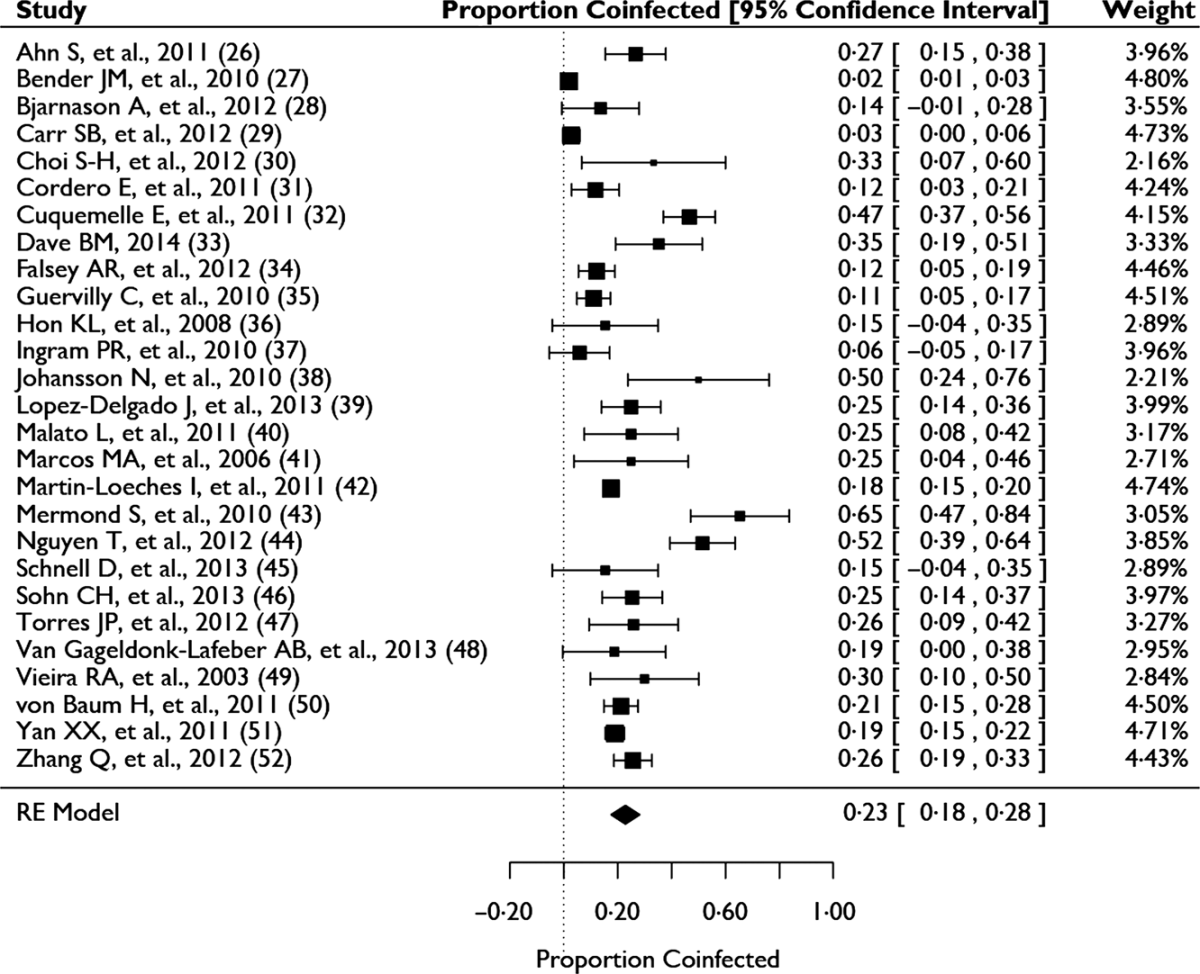
Frequency of bacterial coinfection in hospitalized patients with laboratory confirmed influenza.

Among the individual studies, the proportion of bacterial coinfection ranged from 2% (newborns born in USA) to 65% (immunocompetent adults in France) (Figure 3). High statistical heterogeneity of the included studies (*I* ^*2*^ = 95%) removed confidence in reporting an estimate of the pooled mean proportion of bacterial coinfection through meta‐analysis. Using Cook's distances to identify studies that most greatly affected the heterogeneity and results, we found that seven studies contributed more than 50% of the heterogeneity.^29^, ^31^, ^34^, ^39^, ^40^, ^45^, ^46^ The proportion of bacterial coinfection among the remaining 20 studies, representing 64% of all patients, was between 11% and 35% (*I* ^*2*^ = 37%). Subanalyses and meta‐regression of age, setting, year of enrollment, study type, study size, and bacterial collection method were unable to determine the main sources of heterogeneity. Although heterogeneity was greater in the pediatric studies (*I* ^*2*^ = 98%) than in the adult studies (*I* ^*2*^ = 89%), coinfection frequency was statistically the same (*P *= 0·47). No significant trend was seen when stratifying the studies by mean or median age (Figure S1). Patients enrolled in the ICU had a slightly higher frequency of coinfection than patients enrolled elsewhere, although this was also not statistically significant (*P *= 0·14). Despite the fact that the study period included the 2009 H1N1 pandemic, we observed no significant effect of enrollment year on the frequency of coinfection (*P *= 0·19, Figure S2). The largest study contributed greatly to the heterogeneity of the results; however, study size did not significantly contribute to the heterogeneity in the results (*P *= 0·06 with the largest study, while *P *= 0·41 excluding this study). Finally, study design (*P *= 0·47) and bacterial coinfection collection and detection methods (each was tested independently and all *P*‐values were greater than 0·05) were also not significant. A multivariate analysis also found no significant correlation between any of the variables and overall rates of coinfection.

Several factors may have contributed to heterogeneity between the selected studies that we were unable to quantify. Only four studies explicitly measured and reported on comorbidities of patients with coinfection and found that older age,^28^, ^44^, ^53^ a higher APACHE II (Acute Physiology and Chronic Health Evaluation II) score,^44^, ^53^ diabetes,^33^ and sepsis^33^ were risk factors for coinfection. Further, some studies only included severely immunocompromised patients who had concurrent malignancy or organ transplant,^31^, ^33^, ^49^ while others excluded immunocompromised patients. Severity of illness however was not a factor that could be standardized among the studies.

Antibiotic use at the time of, or prior to enrollment, was another factor that may have contributed to significant heterogeneity, but could not be systematically assessed. Only three studies excluded participants based on antibiotic use and reported coinfection rates of 12·2%, 26·7%, and 46·6%.^28^, ^34^, ^36^ An additional six studies reported on participant antibiotic use at the time of enrollment and found that 12–50% of patients had preceding antibiotic treatment which may have led to an underestimation of the true frequency of bacterial coinfection in their sample population.^30^, ^33^, ^40^, ^45^, ^47^, ^52^ For example, Bjarnason and colleagues found that none of the patients using antibiotics were in the coinfected group and when they excluded antibiotic users from the study, the prevalence of coinfection increased from 14% to 45%.^30^ One study found that previous antibiotic use made patients more likely to acquire atypical bacterial infections.^50^

### Species‐level analysis

**Figure 4:**
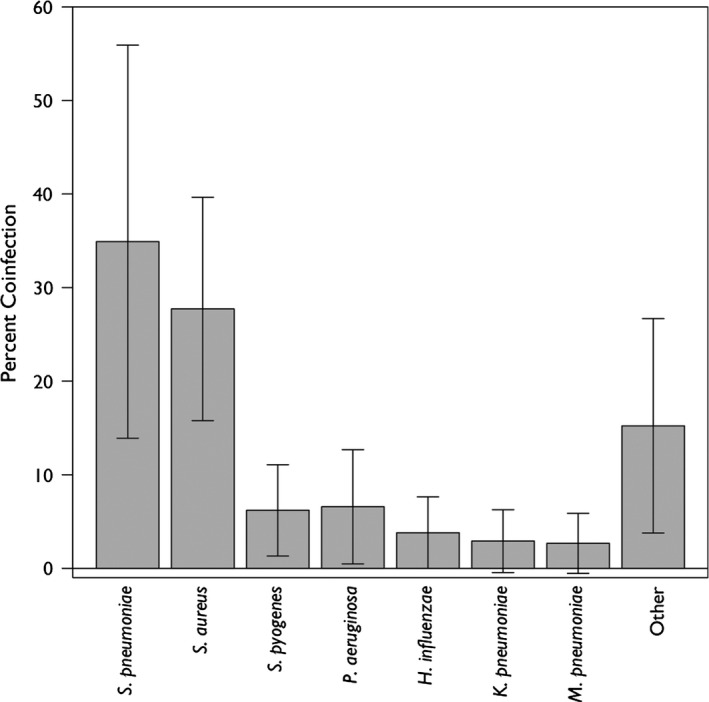
Percent of laboratory confirmed influenza infections that were coinfected by each bacterial species.

*Streptococcus pneumoniae* and *Staphylococccus aureus* were the most common pathogens accounting for 35% (95% CI, 14%–56%) and 28% (95% CI, 16%–40%) of identified coinfecting bacteria, respectively (Figure 4). A number of other pathogens were also identified as causing coinfections: *Pseudomonas aeruginosa, Streptococcus pyogenes*,* Haemophilus influenzae*,* Klebsiella pneumoniae,* and *Mycoplasma pneumoniae*. In addition, other Staphylococcal pathogens such as *S. epidermidis*, and Gram‐negative bacteria, such as *Escherichia coli* and *Moraxella catarrhalis*, were also frequently found.

## Discussion

Despite the long historical understanding of the risk posed by bacterial coinfections in influenza patients,^2^ the extent of coinfection has not been systematically examined.^55^ Understanding the risk of bacterial coinfection in hospitalized patients with influenza can help clinicians balance the need to minimize patient morbidity and mortality due to bacterial infection as well as the individual and societal risks of unnecessary antibiotic use.^12^ To assess the frequency of bacterial coinfection in laboratory confirmed influenza patients, we performed a systematic review and meta‐analysis of papers published since 1982. We found 27 studies covering 3215 patients. The results from these studies were highly variable, ranging from 2% to 65%. Although the majority of studies ranged between 11% and 35%, no specific characteristics of the studies were associated with variability in coinfection frequency. However, there was some suggestion that negative findings or low levels of bacterial coinfection were not published.

Differentiating viral from bacterial infection remains a challenge for clinicians. This diagnostic uncertainty has contributed to a widely recognized overuse of antibiotics in patients with viral illness.^56^, ^57^ The CDC recommends simultaneous antiviral and antibiotic use in the event of influenza‐related pneumonia or suspected bacterial coinfection in patients with influenza.^58^ However, as previous observational studies have shown, patients admitted to the hospital with influenza are more likely to receive antibiotics than antiviral medications.^59^, ^60^ Our findings suggest that while patients hospitalized with moderate to severe influenza may be coinfected with both viral and bacterial pathogens, many patients will likely not be coinfected. Thus, although recognition and treatment of potential bacterial coinfections is important, particularly community‐acquired pneumonia in which pathogens are difficult to detect,^61^ clinicians should consider treatment of potential underlying viral processes as well, particularly for high‐risk patients.^60^ Furthermore, to avoid overuse of antibiotics, our study suggests that routine cultures are advisable in patients hospitalized with influenza, particularly those started on antibiotic therapy empirically. Antibiotic therapy may then be de‐escalated as necessary based on microbiological results.

Consistent with the prior literature,^55^, ^62^ we found that *S. pneumoniae* was the most frequent bacterial coinfection; however, both *S. aureus* and other bacterial coinfections were also quite common. This diverse profile of coinfecting pathogens confirms current Infectious Disease Society of America (IDSA) recommendations for broad‐spectrum antibiotic coverage for influenza‐related pneumonia.^63^ However, although there have been significant increases in the incidence of MRSA infections in the last decade, particularly community‐associated MRSA (CA‐MRSA),^64^ there was not enough data to draw any inferences regarding temporal changes in the etiology of coinfecting pathogens. Given that over 25% of identified isolates were *S. aureus*, and that approximately 50% of hospital *S. aureus* isolates are MRSA,^64^ our study supports IDSA recommendations for empiric coverage of CA‐MRSA in influenza‐related pneumonia patients.^63^

The lack of a statistically significant study covariate may be due to some of the limitations of the study. First of all, although our final sample size included 27 studies and more than 3000 patients, these are relatively small numbers compared to annual estimates of up to 200 000 influenza‐related hospitalizations.^65^ Second, studies included only patients who were hospitalized for influenza and thus represent a population with moderate to severe influenza. Although a few studies enrolled patients in the outpatient or ED setting, all required hospitalization. The study does not, therefore, represent the vast majority of influenza patients, including asymptomatic patients, who are not hospitalized. This identifies a gap in the current literature as the frequency of bacterial coinfection in outpatients with confirmed influenza remains unknown. Third, studies included in this analysis detected bacterial pathogens in a number of different ways, which have varying sensitivity for different organisms and potential coinfecting sites (e.g., sputum versus blood). Some difficult‐to‐detect bacteria may, therefore, be underdiagnosed. Hence, these results may underrepresent the actual number of bacterial coinfections and the distribution of the pathogens of those coinfections. Results for bacterial distribution and likelihood of coinfection may also be affected by colonization rather than infection; however, studies were selected that specifically looked for bacterial coinfection and thus the issue of colonization should be minimal. Finally, we were unable to explain the significant heterogeneity among studies. It was not accounted for by differences in patient age, year, study enrollment setting, study design, study size, or method of bacterial sample collection or detection. This lack of statistically significant variability may be due to unrecorded differences in the studies, such as genetic differences in the populations, local differences in either the severity of viral or bacterial illness, unrecorded patient comorbidities, variation in treatment, or as noted above, antibiotic use (either current or past).

The high heterogeneity and lack of statistically significant covariates also points to the need for additional studies aimed at better understanding rates of bacterial coinfection, outcomes by pathogen, the effect of increased testing for both bacterial and viral pathogens, and the efficacy of interventions, such as increased use of antiviral drugs. These are particularly important in light of recent findings that viral pathogens were more commonly found than bacterial pathogens in suspected community‐acquired pneumonia infections.^61^

## Conclusion

We found that bacterial coinfection of hospitalized patients with influenza is often common, although results were highly heterogeneous. The predominant coinfecting organism in the studies was *S. pneumoniae* followed by *S. aureus*, but many other organisms were also found to cause infections. Providers should consider possible bacterial coinfection in patients hospitalized with influenza, and bacterial cultures should be taken to avoid patient exposure to the risks of prolonged unnecessary antibiotic use. If antibiotic treatment is started, possible coinfection with MRSA should be considered, particularly for community‐acquired pneumonia infections, when selecting appropriate antibiotics, and therapy should be discontinued or de‐escalated as indicated by microbiological results. Finally, the frequency of coinfection should be better characterized in the entire influenza patient population, including outpatients, in future analyses.

## Supporting information


**Table S1** Search terms.
**Table S2** Quality assessment.
**Figure S1** Meta‐regression analysis of patient age on co‐infection frequency.
**Figure S2** Meta‐regression analysis of enrollment year on co‐infection frequency.
