# Supplementary material for: The frequency of influenza and bacterial coinfection: a systematic review and meta‐analysis
Source: Influenza Other Respir Viruses. 2016 Jun 24;10(5):394–403. doi: 10.1111/irv.12398 (PMC4947938; doi:10.1111/irv.12398)
Supplement: Supplementary file 1 — Table S1 Search terms. Table S2 Quality assessment. Figure S1 Meta‐regression analysis of patient age on co‐infection frequency. Figure S2 Meta‐regression analysis of enrollment year on co‐infection frequency. [file IRV-10-394-s001.docx]

**Supplementary Information**

**for**

**The Frequency of Influenza and Bacterial Co-infection: A Systematic Review and Meta-Analysis**

| **Supplementary Table 1: Search Terms** | | | | | |
| --- | --- | --- | --- | --- | --- |
| Concepts | Pubmed/Cochrane | | Embase | | Scopus/Web of Science |
|  | MeSH | Keywords | Emtree | Keywords | Keywords |
| Influenza | influenza, human | human influenza; influenza; flu; influenzas; grippe; H1N1; HINI; HIN1; H1NI; pandemic influenza; 2009 H1N1 influenza; influenza virus; seasonal influenza; influenza virus A H1N1; swine influenza; swine flu; avian influenza; bird flu; Pandemic (H1N1) 2009 Influenza | pandemic influenza; 2009 H1N1 influenza; influenza virus; seasonal influenza; influenza virus A H1N1; swine influenza; avian influenza | human influenza; influenza; influenzas; grippe; flu; H1N1; HINI; HIN1; H1NI; pandemic influenza; 2009 H1N1 influenza; influenza virus; seasonal influenza; influenza virus A H1N1; swine influenza; swine flu; avian influenza; bird flu; pandemic (H1N1) 2009 influenza | human influenza; influenza; flu; influenzas; grippe; H1N1; HINI; HIN1; H1NI; pandemic influenza; 2009 H1N1 influenza; influenza virus; seasonal influenza; influenza virus A H1N1; swine influenza; swine flu; avian influenza; bird flu; Pandemic (H1N1) 2009 Influenza |
| Other infection | Bacterial Infections; Pneumonia, Bacterial | bacterial infection; bacterial infections; bacterial co-infection; bacterial coinfection; bacterial co-infections; bacterial coinfections; bacterial pathogen; bacterial pathogens; bacteremia; bacterial pneumonia; bacterial; bacterial-viral infection; bacterial-viral infections; viral-bacterial infection; viral-bacterial infections | bacterial infection; bacteremia; bacterial pneumonia | bacterial infection; bacterial infections; bacterial co-infection; bacterial coinfection; bacterial co-infections; bacterial coinfections; bacterial pathogen; bacterial pathogens; bacteremia; bacterial pneumonia; bacterial*; bacterial-viral infection; bacterial-viral infections; viral-bacterial infection; viral-bacterial infections | bacterial infection; bacterial infections; bacterial co-infection; bacterial coinfection; bacterial co-infections; bacterial coinfections; bacterial pathogen; bacterial pathogens; bacteremia; bacterial pneumonia; bacterial; bacterial-viral infection; bacterial-viral infections; viral-bacterial infection; viral-bacterial infections |
| Co-infection | Coinfection | Coinfection; co-infection; coinfections; co-infections; co infection; co infections; secondary infection; secondary infections; mixed infection; concomitant infection; concomitant infections; coinfected; co-infected; non-viral co-infection; nonviral coinfection; concomitant; dual infection; dual infections | secondary infection; mixed infection | Coinfection; co-infection; coinfections; co-infections; co infection; co infections; secondary infection; secondary infections; mixed infection; mixed infections; concomitant infection; concomitant infections; coinfected; co-infected; non-viral co-infection; nonviral coinfection; concomitant; dual infection; dual infections | Coinfection; co-infection; coinfections; co-infections; co infection; co infections; secondary infection; secondary infections; mixed infections; mixed infection; concomitant infection; concomitant infections; coinfected; co-infected; non-viral co-infection; nonviral coinfection; concomitant; dual infection; dual infections |
| All terms within each concept combined with OR, all concepts combined with AND. All Pubmed keyword terms searched within textword fields. Cochrane keyword terms were searched within title, abstract and keyword fields. Scopus terms searched within title/abstract/keyword fields. Web of Science terms searched in Topic field. The Cochrane human filter was used in Pubmed and Embase. | | | | | |


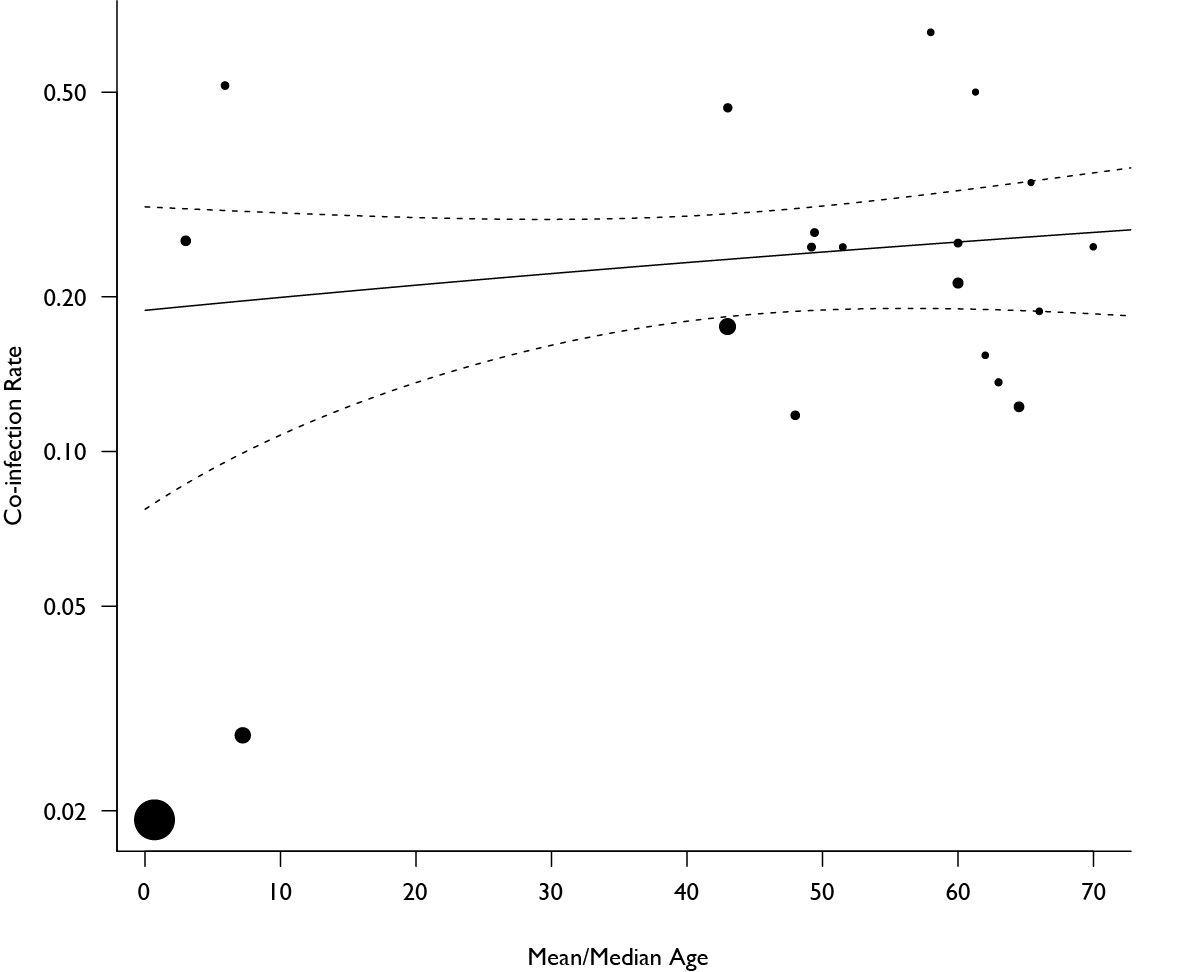


**Supplementary Figure 1**. Meta-regression analysis of patient age on co-infection frequency. Twenty of the studies reported either the mean (10) or the median (10) age of the patients (no study reported both). Analysis used either the mean or the median reported by the study in the regression analysis. Size of the circles represents their weight in the analysis. Despite large weight of study of infants, there does not appear to be a significant relationship between age and co-infection frequencies in patients hospitalized with laboratory confirmed influenza. Similar analysis for mean and median separately found the same qualitative result.


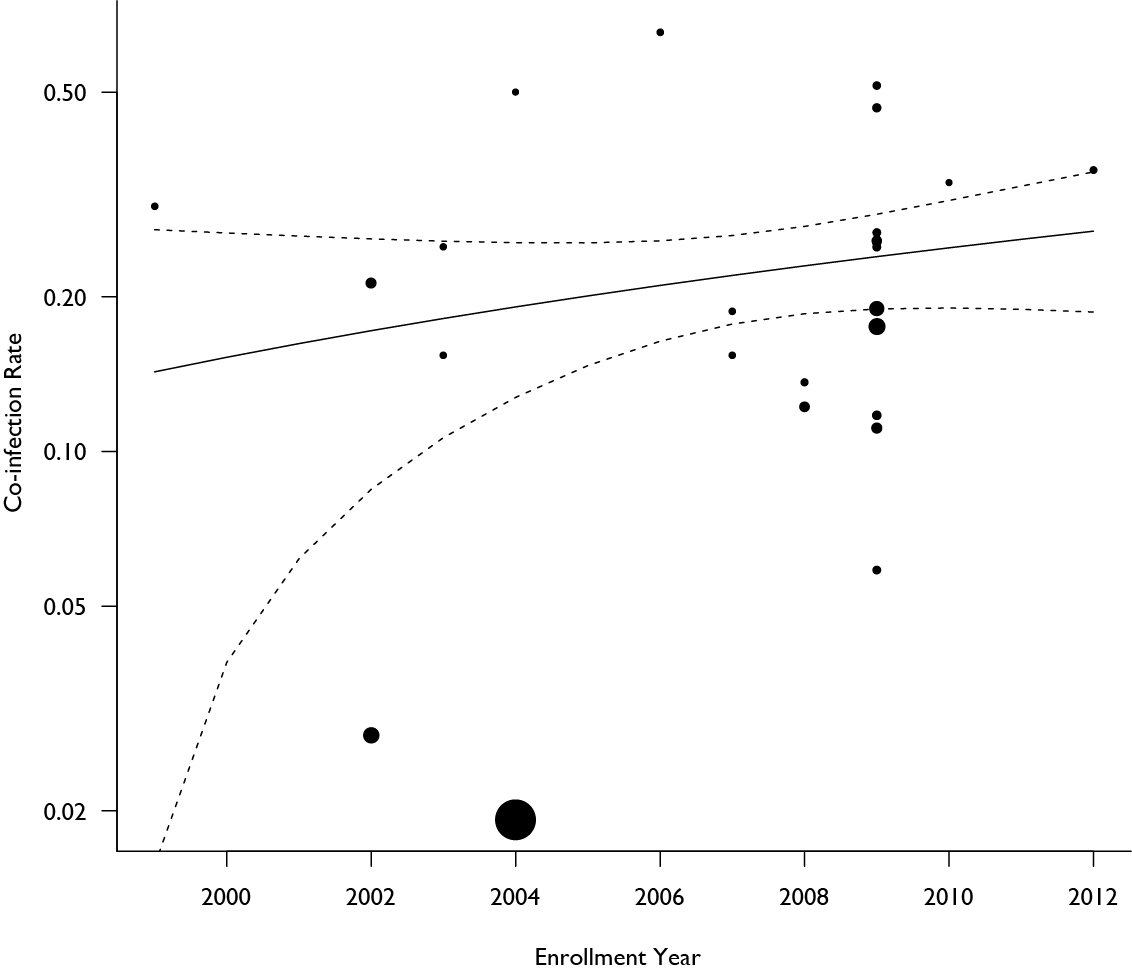


**Supplementary Figure 2**. Meta-regression analysis of enrollment year on co-infection frequency. No significant relationship is apparent between enrollment year and co-infection frequencies. This is significant, as the study encompasses a number of studies from the 2009 H1N1 pandemic.

| **Supplementary Table 2: Quality Assessment** | | | | | | | |
| --- | --- | --- | --- | --- | --- | --- | --- |
|  | Selection Bias | Study Design | Confounders | Blinding | Data Collection Methods | Withdrawals | Global |
| Ahn S, et al. 2011 [[26](#_ENREF_26)] | M | M/W | S | S | S | S | Strong |
| Bender JM, et al. 2010 [[27](#_ENREF_27)] | M | M | S/M | M | S/M | S | Moderate |
| Bjarnason A, et al. 2012 [[28](#_ENREF_28)] | M | M | S/M | S/M | S | M | Moderate |
| Carr SB, et al. 2012 [[29](#_ENREF_29)] | S | M | S/M | S | W | M | Moderate |
| Choi S-H, et al. 2012 [[30](#_ENREF_30)] | S | M | M | M | M | S | Moderate |
| Cordero E, et al. 2011 [[31](#_ENREF_31)] | M | M/W | M | S | S | S | Moderate |
| Cuquemelle E, et al. 2011 [[32](#_ENREF_32)] | M | M | M | S | S/M | S | Moderate |
| Dave BM 2014 [[33](#_ENREF_33)] | S | M/W | S | M | S | S | Strong |
| Falsey AR, et al. 2012 [[34](#_ENREF_34)] | S | M | W | S | S | S | Moderate |
| Guervilly C, et al. 2010 [[35](#_ENREF_35)] | M | M | M | S | M | M | Moderate |
| Hon KL, et al. 2008 [[36](#_ENREF_36)] | S/M | W | S | S | W | S | Weak |
| Ingram PR, et al. 2010 [[37](#_ENREF_37)] | W | M/W | S | S | W | S | Weak |
| Johansson N, et al. 2010 [[38](#_ENREF_38)] | S/M | M/W | S | S | S | S | Strong |
| Lopez-Delgado J, et al. 2013 [[39](#_ENREF_39)] | M | M | M | S | M | W | Moderate |
| Malato L, et al. 2011 [[40](#_ENREF_40)] | S/M | M/W | N/A | S | M | S | Moderate |
| Marcos MA, et al. 2006 [[41](#_ENREF_41)] | M | M | S/M | S | S | S | Moderate |
| Martin-Loeches I, et al. 2011 [[42](#_ENREF_42)] | M | M | M | S | S | S | Moderate |
| Mermond S, et al. 2010 [[43](#_ENREF_43)] | M | M | S | S | S/M | S | Moderate |
| Nguyen T, et al. 2012 [[44](#_ENREF_44)] | M | M | M/W | S | S | M | Moderate |
| Schnell D, et al. 2013 [[45](#_ENREF_45)] | M | M/W | W | S | S | S | Moderate |
| Sohn CH, et al. 2013 [[46](#_ENREF_46)] | M | M | M | S | S | S/M | Moderate |
| Torres JP, et al. 2012 [[47](#_ENREF_47)] | S | M | M | S | W | S | Moderate |
| Van Gageldonk-Lafeber AB, et al. 2013 [[48](#_ENREF_48)] | S | M/W | W | N/A | S | S | Moderate |
| Vieira RA, et al. 2003 [[49](#_ENREF_49)] | W | M | M | S | W | W | Weak |
| von Baum H, et al. 2011 [[50](#_ENREF_50)] | S | M | M | S | S | S | Strong |
| Yan XX, et al. 2011 [[51](#_ENREF_51)] | W | W | M | S | S/M | W | Weak |
| Zhang Q, et al. 2012 [[52](#_ENREF_52)] | S | M | S/M | S | S | S | Strong |
| S = Strong; S/M = One author gave a rating of strong, and one gave a rating of moderate; M = Moderate; M/W = One author gave a rating of moderate, and one gave a rating of weak; W = Weak. Colors are merely for helping to differentiate the results. | | | | | | | |
